# A Hierarchical PSMC–LQR Control Framework for Accurate Quadrotor Trajectory Tracking

**DOI:** 10.3390/s25227032

**Published:** 2025-11-18

**Authors:** Shiliang Chen, Xinyu Zhu, Yichao Fang, Yucheng Zhan, Dan Han, Yun Qiu, Yaru Sun

**Affiliations:** 1 Institute of Electronic and Electrical Engineering, Civil Aviation Flight University of China, 46 Nanchang Road, Guanghan 618307, China; shiliang_ccc@163.com (S.C.); cafuczxy@dingtalk.com (X.Z.); fyc2934844531@163.com (Y.F.); qq1097188704@163.com (Y.Z.); yuky_qy@163.com (Y.Q.); syr130826@163.com (Y.S.); 2 University of Chinese Academy of Sciences, Beijing 100049, China; 3 Institute of Electrical Engineering Chinese Academy of Sciences, Beijing 100190, China

**Keywords:** trajectory tracking, nonlinear system, disturbance rejection control, model predictive control, particle swarm optimization, linear quadratic regulator

## Abstract

Accurate trajectory tracking of quadrotor UAVs remains challenging due to highly nonlinear dynamics, model uncertainties, and time-varying external disturbances, which make it difficult to achieve both precise position tracking and stable attitude regulation under control constraints. To tackle these coupled problems, this paper develops a hierarchical control framework in which the outer-loop particle swarm optimization (PSO)-compensated model predictive controller (PSMC) adaptively mitigates prediction errors and enhances robustness, while the inner-loop enhanced linear quadratic regulator (LQR), augmented with gain scheduling and control-rate relaxation, accelerates attitude convergence and ensures smooth control actions under varying flight conditions. A Lyapunov-based stability analysis is conducted to ensure closed-loop convergence. Simulation results on a helical reference trajectory show that, compared with the conventional MPC–LQR baseline, the proposed framework reduces the mean tracking errors by more than 13.2%, 17.1%, and 28% in the x-, y-, and z-directions under calm conditions, and by more than 34%, 26.2%, and 46.8% under wind disturbances. These results prove that the proposed hierarchical PSMC–LQR framework achieves superior trajectory tracking accuracy, strong robustness, and high practical implement ability for quadrotor control applications.

## 1. Introduction

In recent decades, significant progress has been made in the development of quadrotor unmanned aerial vehicles (UAVs). Owing to their low cost, compact size, lightweight structure, high maneuverability, vertical take-off and landing (VTOL) capability, enhanced payload capacity, and ability to operate in harsh environments, UAVs have gained widespread popularity [1]. The quadrotor UAV is a typically underactuated and strongly coupled system with four inputs and six outputs, making it an underactuated system [2]. In the design of its control system, position and attitude dynamics are usually decoupled using a hierarchical control structure, where the outer loop is responsible for position control and the inner loop for attitude stabilization. However, during actual flight, a quadrotor is often subjected to various unknown disturbances, which pose significant challenges to controller design. External disturbances can cause instability or even divergence in the control system, potentially resulting in severe damage to the UAV. Therefore, improving the disturbance rejection capability of a quadrotor is crucial not only for achieving precise trajectory tracking but also for ensuring flight safety [3].

To address the trajectory tracking problem of quadrotor UAVs, several advanced control strategies have been developed, including proportional-integral-derivative (PID) control [4], robust control [5], sliding mode control (SMC) [6,7,8], and backstepping control (BC) [9,10]. However, some of these approaches lack robustness against external disturbances, while others achieve robustness at the expense of control performance. In addition, several robust control approaches have been developed to simultaneously address disturbances and uncertainties [11,12]. These works provide valuable insights into compensation mechanisms under uncertain dynamics. Recent improvements such as adaptive fractional-order sliding modes, disturbance-observer enhanced SMC, and finite-time convergence methods yield better robustness but still often suffer from chattering, limited convergence speed, or sensitivity to gain tuning [13,14]. Hybrid control strategies and learning-based flight control theories, such as neural networks [15] and fuzzy logic [16], have also been developed for quadrotor UAV control. These control methods offer flexibility and adaptability for quadrotor UAV control but suffer from several notable drawbacks. Neural network controllers typically lack formal stability guarantees, require large and representative training datasets, and involve complex tuning of hyperparameters, making them difficult to generalize and validate. Fuzzy logic controllers, on the other hand, heavily rely on expert-defined rules and membership functions, lack rigorous stability analysis, and often exhibit limited tracking precision and high computational cost. These limitations hinder their reliability in high-precision and safety-critical UAV applications [17,18].

MPC has been widely applied to UAV trajectory tracking due to its ability to predict future behavior based on reference information, explicitly handle system constraints, and leverage well-established theoretical frameworks that provide guarantees on closed-loop performance [19]. A common feature in many quadrotor control approaches is the use of a cascaded control architecture, where attitude control (inner loop) is decoupled from position and velocity control (outer loop) [20]. In ref. [21], a strategy combining a thrust-prioritized inner-loop controller with an outer-loop controller that satisfies certain state constraints is proposed; however, it also lacks formal guarantees of stability and convergence. To overcome the limitations of designing controllers for highly nonlinear systems such as quadrotors using linear representations, nonlinear model predictive control (NMPC) strategies have been extensively employed [22]. In ref. [23], NMPC is utilized to avoid obstacles in cluttered environments while executing high-speed trajectories, considering both input and state constraints. In ref. [24], NMPC is compared with differential flatness-based control (DFBC) using an incremental nonlinear dynamic inversion method. Results, with and without an inner-loop controller, show that NMPC achieves better performance than DFBC, at the expense of higher computational cost. While these methods show impressive high-speed tracking performance in real experiments, they lack formal stability guarantees, leading to failures during aggressive maneuvers. Additionally, the feasibility of the NMPC algorithm is not ensured, causing potentially unpredictable and unstable behavior.

LQR has been effectively applied to a wide range of complex systems, such as double-inverted pendulums, fuel cell systems, vibration control systems, and electric vehicles. It has also been widely adopted in robotic trajectory tracking control [25,26,27]. The quadratic cost function of the LQR controller consists of two weighting matrices, namely the Q and R matrices. The Q matrix is associated with the trajectory deviation of the state variables, while the R matrix relates to control effort and actuator saturation. A key challenge in the real-time application of the LQR optimal controller lies in the effective selection of the Q and R weighting matrices [28], which inherently involves trade-offs and is typically tuned through a trial-and-error process.

Despite the significant progress in quadrotor control, three major challenges still hinder accurate trajectory tracking in practical scenarios. First, the inherently nonlinear and strongly coupled dynamics complicate controller design and make linear assumptions insufficient for aggressive or fast maneuvers [29]. Second, external disturbances and model uncertainties, such as aerodynamic effects, unmodeled dynamics, and time-varying flight conditions, can severely degrade tracking performance if not properly compensated [30]. Third, state and input constraints impose additional limitations on controller execution, particularly when rapid response and smooth control allocation are required [31]. These issues motivate the development of a hierarchical control framework that can simultaneously address prediction accuracy, robustness enhancement, and attitude stabilization within a unified structure.

To address the limitations of existing methods, this paper proposes a hierarchical PSMC–LQR control framework. A PSO-based compensation mechanism is integrated into the outer-loop MPC to correct prediction mismatches and enhance robustness against model uncertainties and disturbances. In the inner loop, an LQR controller is augmented with gain scheduling and control-rate relaxation to improve attitude convergence and smooth actuation under varying flight conditions. This hierarchical design leverages the predictive optimization ability of MPC, the adaptive error correction of the PSO-based compensator, and the fast stabilization of the enhanced LQR. In addition, the proposed framework ensures closed-loop stability through Lyapunov-based analysis and maintains moderate computational complexity suitable for practical deployment. The main contributions of this work are summarized as follows:

- A hierarchical control architecture is developed, in which a model predictive controller is coupled with a PSO-based compensator in the outer loop, and an enhanced LQR with gain scheduling and rate relaxation is employed in the inner loop. This structure decouples position and attitude control while improving adaptability to time-varying disturbances.
- A PSO-compensated MPC (PSMC) scheme is designed to correct prediction mismatches and suppress external perturbations. By iteratively optimizing compensation terms based on tracking error feedback, the outer-loop control improves robustness without increasing the MPC horizon or computational load.
- An inner-loop LQR is augmented with gain scheduling and control-rate relaxation to enhance attitude convergence and reduce actuator fluctuation. The former adapts feedback gains online according to the system state, while the latter smooths control updates to avoid excessive rate changes. This combination accelerates tracking response and improves stability under uncertain conditions.

We have organized the paper as follows. In Section 2, the nonlinear dynamics of the quadrotor are introduced, and a discrete-time, control-oriented model is derived for use in predictive control. Section 3 presents the proposed hierarchical PSMC–LQR control framework, including the outer-loop PSMC-based position controller and the inner-loop LQR-based attitude stabilizer. In Section 4, the closed-loop stability of the proposed hierarchical controller is analyzed using a discrete-time Lyapunov framework. In Section 5, extensive simulation results are provided to evaluate tracking performance, disturbance rejection, and computational complexity of the proposed method in comparison with two baseline schemes. In Section 6, concluding remarks are drawn and directions for future work are discussed.

## 2. System Modeling and Problem

### 2.1. Quadrotor Dynamics

**Figure 1:**
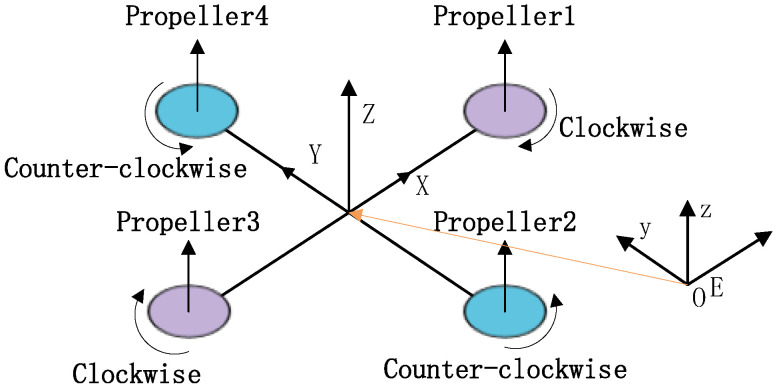
The body frame and the inertial (Earth) frame, where “E” denotes the origin of the inertial frame.

A quadrotor helicopter is a type of multi-rotor aircraft equipped with four fixed-pitch propellers mounted symmetrically on a cross-shaped frame, as illustrated in Figure 1. Propeller pairs (1, 3) rotate clockwise, while pairs (2, 4) rotate counterclockwise, which ensures that the overall rotational torque is balanced [32]. Additionally, the thrust generated by each propeller is aligned in the same upward direction, contributing to stable lift generation. Based on the structural characteristics of the quadrotor, the following assumptions are made [33]:

1. The quadrotor is modeled as a rigid body with constant mass and moment of inertia.
2. The center of mass of the UAV remains fixed and coincides with the geometric center of the frame.
3. The UAV is subjected only to gravitational force and thrust generated by the propellers, while aerodynamic drag is neglected.

Based on the Newton–Euler method, the nonlinear dynamic model of the quadrotor UAV is formulated as follows:

(1) $$\left\{\begin{array}{l} \ddot{\phi }=\frac{I_{y}-I_{z}}{I_{x}}\dot{\theta }\dot{\psi }+\frac{I_{r}}{I_{x}}\dot{\theta }\omega_{\mathrm{sum}}+\frac{1}{I_{x}}U_{2}\\ \ddot{\theta }=\frac{I_{z}-I_{x}}{I_{y}}\dot{\phi }\dot{\psi }-\frac{I_{r}}{I_{y}}\dot{\phi }\omega_{\mathrm{sum}}+\frac{1}{I_{y}}U_{3}\\ \ddot{\psi }=\frac{I_{x}-I_{y}}{I_{z}}\dot{\phi }\dot{\theta }+\frac{1}{I_{z}}U_{4}\\ \ddot{x}=-\frac{U_{1}}{m}\left(\sin\theta \cos\phi \cos\psi +\sin\phi \sin\psi \right)\\ \ddot{y}=-\frac{U_{1}}{m}\left(\sin\theta \cos\phi \sin\psi -\sin\phi \cos\psi \right)\\ \ddot{z}=-\frac{U_{1}}{m}\cos\theta \cos\phi +g \end{array}\right.$$

In the equations, ϕ,θ, and ψ represent the roll, pitch, and yaw angles of the quadrotor, respectively; x, y and z denote the quadrotor’s position in the three-dimensional Cartesian space. ${I_{x},{\;I}_{y},I}_{z}$ are the moments of inertia of the UAV, respectively, and $I_{r}$ is the rotational inertia of the rotor, g denotes the acceleration due to gravity. $\omega_{\mathrm{sum}}\;$ is the algebraic sum of the angular velocities of the four rotors, defined as $\omega_{\mathrm{sum}}=\omega_{1}-\omega_{2}+\omega_{3}-\omega_{4}$, where $\omega_{1},{\;\omega }_{2},{\;\omega }_{3},{\;\omega }_{4}$ are the rotational speeds of the four propellers. $U_{1},{\;U}_{2},{\;U}_{3},{\;U}_{4}$ represent the total thrust, roll torque, pitch torque, and yaw torque generated by the UAV, respectively, and are defined as follows:

(2) $$\left\{\begin{array}{l} U_{1}=b\left(\omega_{1}^{2}+\omega_{2}^{2}+\omega_{3}^{2}+\omega_{4}^{2}\right)\\ U_{2}=lb\left(-\frac{\sqrt{2}}{2}\omega_{1}^{2}+\frac{\sqrt{2}}{2}\omega_{2}^{2}+\frac{\sqrt{2}}{2}\omega_{3}^{2}-\frac{\sqrt{2}}{2}\omega_{4}^{2}\right)\\ U_{3}=lb\left(\frac{\sqrt{2}}{2}\omega_{1}^{2}-\frac{\sqrt{2}}{2}\omega_{2}^{2}+\frac{\sqrt{2}}{2}\omega_{3}^{2}-\frac{\sqrt{2}}{2}\omega_{4}^{2}\right)\\ U_{4}=d\left(\omega_{1}^{2}+\omega_{2}^{2}-\omega_{3}^{2}-\omega_{4}^{2}\right) \end{array}\right.$$

where b is the thrust coefficient of the rotor, d is the drag coefficient of the rotor, and l is the distance from the rotor axis to the quadrotor’s center of mass.

### 2.2. Problem Formulation

To formalize the control problem, consider a quadrotor system represented by a continuous nonlinear state-space model of the form:

(3) $$\dot{x}\left(t\right)=f\left(x\left(t\right),u\left(t\right)\right)+d\left(t\right)$$

where $x\left(t\right)\in R^{6}$ is the state vector comprising position and velocity components in the inertial frame, $u\left(t\right)\in R^{3}$ is the control input (translational force vector), and $d\left(t\right)\in R^{6}$ represents unknown but bounded external disturbances. In this paper, the external disturbance term d(t) represents physical effects such as wind gusts, airflow fluctuations, and unmodeled aerodynamic forces that act on the translational dynamics of the UAV. These perturbations are introduced into the system as bounded additive forces and later instantiated as harmonic wind disturbances in Section 5.

Let the desired trajectory be defined by a smooth reference signal:

(4) $$\zeta_{d}\left(t\right)={\left[x_{d}\left(t\right)\begin{array}{ccc} \begin{array}{cc} \begin{array}{cc} \begin{array}{c} {\;\;y}_{d}\left(t\right) \end{array} & z_{d}\left(t\right) \end{array} & {\dot{x}}_{d}\left(t\right) \end{array} & {\dot{y}}_{d}\left(t\right) & {\dot{z}}_{d}\left(t\right) \end{array}\right]}^{T}$$

The tracking error is defined as:

(5) $$e\left(t\right)=\zeta \left(t\right)-\zeta_{d}\left(t\right)$$

The discrete-time error dynamics, obtained via zero-order hold discretization with sampling interval Δt, can be expressed as:

(6) $$e_{k+1}=A_{d}e_{k}+B_{d}u_{k}+d_{k}$$

where $A_{d}$ and $B_{d}$ are the linearized and discretized state-space matrices, and $d_{k}$ denotes the bounded disturbance at time step k. The objective of control is to design a stabilizing controller $u_{k}$ such that:

- The tracking error $e_{k}\to 0$ asymptotically or remains bounded within a small neighborhood.
- State and input constraints are satisfied: $x_{k}\in X$ and $u_{k}\in U$.
- The controller is suitable for real-time embedded implementation.

Mathematically, the problem is formulated as:

(7) $$min\sum_{u_{0},\ldots u_{N-1}}^{N-1}\;{∥e_{k+i}∥}^{2}Q+{∥u_{k+i}∥}^{2}R\;s.t.\;\;\;\;\;\;\;\;\;\;e_{k+i+1}=A_{d}e_{k+i}+B_{d}u_{k+i}+d_{k+i}\;\;\;\;\;\;\;\;\;\;\;\;\;\;\;\;\;\;\;\;\;\;\;x_{k+i}\in X,u_{k+i}\in U,\forall i\in [0,N-1]$$

where Q>0 and R>0 are weight matrices, X and U represent feasible state and input sets, and N is the prediction horizon. This formulation serves as the foundation for the development of the outer-loop PSMC and the inner-loop LQR controller, as elaborated in subsequent sections.

The continuous-time quadrotor model is discretized using zero-order hold assumptions to facilitate predictive control design. The detailed linearization and discretization procedure is provided in Appendix A.

## 3. Hierarchical Control Architecture and Controller Design

### 3.1. Hierarchical Control Architecture

**Figure 2:**
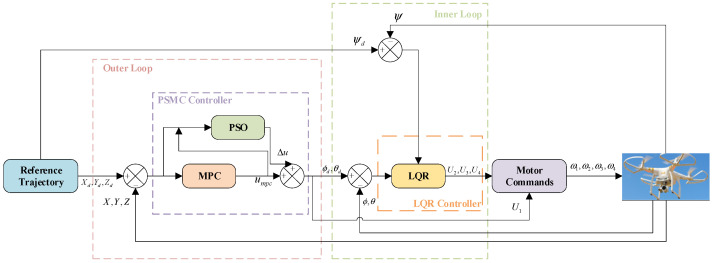
Detailed module-level signal-flow diagram of the hierarchical PSMC–LQR controller.

To achieve real-time and robust trajectory tracking under disturbances and constraints, a hierarchical control architecture is established, consisting of an outer-loop trajectory controller and an inner-loop attitude stabilizer, as illustrated in Figure 2. This cascaded structure follows the principle of time-scale separation: the outer loop operates at a slower update rate to plan motion in the position domain, while the inner loop runs at a faster rate to stabilize attitude dynamics and ensure rapid torque response. Such decomposition not only simplifies controller design for the inherently nonlinear quadrotor system but also enhances robustness and computational efficiency during onboard execution.

In this architecture, the outer loop employs a PSO-compensated MPC framework. Its primary task is to compute optimal thrust and attitude references that minimize position-tracking errors while satisfying input and state constraints. Let the position state vector be $\gamma =[x,y,z{]}^{T}$, and the desired reference trajectory be $\gamma_{d}=[x_{d},y_{d},z_{d}{]}^{T}$. The tracking error is defined as: $e_{k}=\gamma_{k}-\gamma_{d,k}$, and the outer loop solves the following quadratic optimization problem at each sampling instant:

(8) $$u_{MPC}=arg\;\underset{u}{min}\;\sum_{i=0}^{N-1}\;{∥e_{k+i}∥}_{Q}^{2}+{∥u_{k+i}∥}_{R}^{2}$$

subject to discrete-time linearized system dynamics and input/state constraints. To enhance adaptability, a PSO-based compensation module is introduced to dynamically adjust the MPC solution, yielding the refined control signal:

(9) $$u_{k}^{*}=u_{MPC}+\Delta u_{PSO}$$

This optimized control signal is then transformed through inverse kinematics into the desired attitude command $\sigma_{d}=[\phi_{d},\theta_{d},\psi_{d}{]}^{T}$, which serves as the reference input to the inner loop. The inner-loop controller utilizes an LQR to stabilize the quadrotor’s rotational dynamics. Its control law is defined as:

(10) $$\tau =-K_{LQR}(\sigma -\sigma_{d})$$

where $\sigma =[\phi ,\;\theta ,\;\psi {]}^{T}$ is the measured attitude vector, and $K_{\mathrm{LQR}}$ is the gain matrix designed through optimization and augmented with gain scheduling and control-rate relaxation to adapt to different flight regimes. The inner loop ensures fast attitude convergence, smooth actuation, and effective disturbance rejection, which in turn improves the accuracy and stability of the outer-loop trajectory tracking.

In summary, the proposed hierarchical PSMC–LQR architecture establishes a clear division of control responsibilities:

- The outer loop focuses on trajectory planning, constraint handling, and robustness enhancement through adaptive optimization.
- The inner loop guarantees high-bandwidth attitude stabilization and torque control.

The bidirectional information flow—where the outer loop provides desired attitude and thrusts references, and the inner loop returns stabilized attitude feedback—forms a cooperative closed-loop system. This integration achieves precise position tracking, strong disturbance tolerance, and low-latency computation, making the framework highly suitable for real-time embedded flight control.

### 3.2. Outer-Loop Controller

To enhance the effectiveness of the outer-loop position controller, which is implemented using a PSMC framework, the PSO algorithm is introduced as an auxiliary adaptive module. The conventional MPC component provides nominal trajectory optimization, while the PSO compensator performs real-time correction to mitigate prediction errors arising from model inaccuracies and unmodeled disturbances. This cooperative design improves the robustness and adaptability of the outer loop without increasing the prediction horizon or tightening the control constraints. Therefore, PSO is discussed separately in the following subsection to elucidate its optimization mechanism.

#### 3.2.1. Particle Swarm Optimization

PSO algorithm, inspired by the foraging behavior of bird flocks [34], enables a disordered population to evolve into an organized system through information sharing among individuals, thereby searching for feasible solutions in the solution space. In PSO, each potential solution to the optimization problem is represented by a particle in the search space. During each iteration, the fitness value of a particle’s position is evaluated based on the objective function. The particle then updates its velocity and trajectory based on both its own best-known position and the global best-known position discovered by the swarm. Let the number of decision variables be D; then each particle can be represented as a D-dimensional vector. The position and velocity of the ith particle are defined as:

(11) $$X_{i}=\left(x_{i1},x_{i1},\ldots ,x_{iD}\right)$$

(12) $$V_{i}=\left(v_{i1},v_{i1},\ldots ,v_{iD}\right)$$

The particle velocity update equation is given by:

(13) $$\begin{array}{c} v_{id}^{n+1}=\omega \cdot v_{id}^{n}+c_{1}\cdot r_{1}^{n}\left(Pbest_{id}^{n}-x_{id}^{n}\right) \end{array}+c_{2}\cdot r_{2}^{n}\left(Gbest_{id}^{n}-x_{id}^{n}\right)$$

(14) $$\nu_{min}\leq \nu_{id}^{n}\leq \nu_{max}$$

The particle position update equation is given by:

(15) $$x_{id}^{n+1}=x_{id}^{n}+v_{id}^{n+1}$$

In the particle velocity update equation, $v_{id}^{n+1}$ denotes the velocity of the ith particle in the dth dimension at iteration n+1, and $\nu_{id}^{n}$ represents its velocity at iteration n. The values $\nu_{min}$ and $\nu_{max}$ define the lower and upper bounds of the particle velocity, respectively. $Pbest_{id}^{n}$ refers to the best historical position of the ith particle in the dth dimension up to iteration n, while $Gbest_{id}^{n}$ denotes the global best position of the entire swarm up to iteration n.

The swarm consists of N particles, where i=1, 2,…,N, and each particle has D decision variables, indexed by d=1, 2,⋯,D. The parameters $c_{1}$ and $c_{2}$ are cognitive and social learning factors, respectively. $c_{1}$ governs the tendency of a particle to move toward its own best position Pbest, while $c_{2}$ influences movement toward the global best position Gbest. The terms $r_{1}^{n}$ and $r_{2}^{n}$ are random numbers uniformly distributed in the range [0, 1], introduced to provide stochastic behavior in the search process.

The inertia weight ω regulates the trade-off between global and local exploration capabilities. A larger value of ω encourages global exploration and helps the particles escape local optima, whereas a smaller value leads to faster convergence and promotes local search. Typically, ω is selected within the range [0, 1].

In this study, a linearly decreasing strategy is employed to dynamically adjust the inertia weight. A relatively large inertia weight at the early stages of the algorithm enhances the global search capability, while gradually reducing the inertia weight as iterations proceed facilitate faster convergence to the global optimum. The inertia weight is updated according to the following equation:

(16) $$\omega =\omega_{max}-\frac{\omega_{max}-\omega_{min}}{n_{max}}n$$

where $\omega_{max}$ denotes the initial inertia weight, $\omega_{min}$ represents the final inertia weight, and $n_{max}$ is the maximum number of iterations.

The general procedure of the PSO algorithm is summarized in Algorithm 1.
**Algorithm 1.** Particle Swarm Optimization ProcedureInput: Objective function f(x), number of particles N, dimension D, inertia weight ω, acceleration coefficients c1, c2, max iterations $\;T_{max}$
    Initialize particle positions $x_{i}\;\in R^{D}$ and velocities $v_{i}\in R^{D}$ for i = 1 to N
    Initialize personal bests $p_{best}\leftarrow x_{i}$, evaluate $\;f(p_{besti})$
       Set $\;g_{best}\leftarrow {argmin}_{i}\cdot f(p_{besti})$
For t = 1  to $T_{max}\;$ DO
  For  i = 1  to N  DO        Update velocity $v_{i}\leftarrow \omega *v_{i}+c_{1}*rand*\left(p_{besti}-x_{i}\right)+c_{2}*rand*\left(g_{besti}-x_{i}\right)$        Update position $x_{i}\;\leftarrow \;x_{i}\;+\;v_{i}$        Evaluate fitness $f(x_{i})$        If $f(x_{i})\;<\;f(p_{besti})$ then           $p_{besti}\;\leftarrow x_{i}$        If $f(p_{besti})\;<\;f(g_{best})$ then           $g_{best}\;\leftarrow \;p_{besti}$        END IF     END IF   END FOR  END FOR return $g_{best}$, $f\left(g_{best}\right)$ Output: Global best position $g_{best}$ and value $f(g_{best})$

#### 3.2.2. PSMC Outer-Loop Controller

In this section, a PSMC outer-loop controller is developed by integrating an MPC framework with a PSO-based compensator. The MPC component determines the optimal control input at each time step through a combination of model prediction, rolling horizon optimization, and feedback correction, enabling anticipative and constraint-aware control. To enhance the system’s adaptability to dynamic and uncertain environments, the PSO-based compensator evaluates the current quadrotor state in real time and provides online compensation to the MPC-generated control signals. This integrated design improves the controller’s robustness and tracking accuracy under varying operational conditions. In addition to external disturbances, the quadrotor model may also exhibit parametric uncertainties arising from factors such as unmodeled aerodynamics, actuator imperfections, or deviations in physical parameters. In this work, these uncertainties are treated implicitly as part of the overall disturbance input and are not modeled separately. The robustness of the proposed controller against such effects is reflected through the compensative structure of the outer-loop and validated in the simulation results of Section 5.

For the position dynamics model of a quadrotor, the design can be divided into two components: the first involves generating vertical thrust $U_{1}\left(t\right)$ through the rotors; the second involves controlling the UAV’s motion in the x and y directions by adjusting the direction of this thrust. The spatial position state equation of the UAV system is defined as $\xi \left(t\right)=[x(t)\;\dot{x}(t)\;y(t)\;\dot{y}(t)\;z(t)\;\dot{z}(t){]}^{T}$, where $x\left(t\right)$,$\;y\left(t\right)$ and $z\left(t\right)$ represent the position coordinates of the UAV in the inertial (ground-fixed) frame, and $\dot{x}\left(t\right)$, $\dot{y}\left(t\right)$ and $\dot{z}\left(t\right)$ represent the corresponding velocity components. The motion equations can be reformulated as follows:

(17) $$\dot{\xi }\left(t\right)=f\left(\xi \left(t\right),u_{\xi }\left(t\right)\right)=\left[\begin{array}{c} \dot{x}\left(t\right)\\ \frac{U_{1}\left(t\right)}{m}u_{x}\left(t\right)+\frac{d_{fx}\left(t\right)}{m}\\ \dot{y}\left(t\right)\\ \frac{U_{1}\left(t\right)}{m}u_{y}\left(t\right)+\frac{d_{fy}\left(t\right)}{m}\\ \dot{z}\left(t\right)\\ -g+\frac{U_{1}\left(t\right)}{m}\left(\cos\varphi \left(t\right)\cos\theta \left(t\right)\right)+\frac{d_{fz}\left(t\right)}{m} \end{array}\right]$$

where

(18) $$\begin{array}{c} u_{x}\left(t\right)=\cos\psi \left(t\right)\sin\theta \left(t\right)\cos\varphi \left(t\right) \end{array}+\sin\psi \left(t\right)\sin\varphi \left(t\right)$$

(19) $$\begin{array}{c} u_{y}\left(t\right)=\sin\psi \left(t\right)\sin\theta \left(t\right)\cos\varphi \left(t\right) \end{array}-\cos\psi \left(t\right)\sin\varphi \left(t\right)$$

For a given reference trajectory, it is assumed to satisfy the following state equation:

(20) $${\dot{\bar{\xi }}}_{r}\left(t\right)=f\left({\bar{\xi }}_{r}\left(t\right),u_{\xi r}\left(t\right)\right)$$

where ${\bar{\xi }}_{r}(t)=[x_{r}\left(t\right)\;\;{\dot{x}}_{r}\left(t\right)\;y_{r}\left(t\right)\;{\dot{y}}_{r}\left(t\right)\;z_{r}\left(t\right)\;{\dot{z}}_{r}(t){]}^{T}$ denotes the reference state vector, and $u_{\xi r}\left(t\right)={\left[\begin{array}{ccc} u_{xr} & u_{yr} & U_{1r} \end{array}\right]}^{T}$ represents the corresponding reference control input vector. The reference inputs are defined as follows:

(21) $$u_{x_{r}}=\frac{m{\ddot{x}}_{r}\left(t\right)}{U_{1}\left(t\right)},\;\;\;\;U_{1_{r}}\left(t\right)=\frac{m\left({\ddot{z}}_{r}\left(t\right)+g\right)}{\cos\theta \left(t\right)\cos\phi \left(t\right)},\;\;\;\;u_{y_{r}}=\frac{m{\ddot{y}}_{r}\left(t\right)}{U_{1}\left(t\right)}$$

To derive the error dynamics of the system, define the state error vector as $\tilde{\bar{\xi }}\left(t\right)=\bar{\xi }\left(t\right)-{\bar{\xi }}_{r}\left(t\right)$, and the control input error as ${\tilde{u}}_{\xi }\left(t\right)={\tilde{u}}_{\xi }\left(t\right)-{\tilde{u}}_{\xi r}\left(t\right)$. The resulting state-space error dynamics can then be expressed as shown in Equation (17):

(22) $$\overset{˙}{\tilde{\bar{\xi }}}\left(t\right)=A\left(t\right)\tilde{\bar{\xi }}\left(t\right)+B\left(t\right){\tilde{u}}_{\xi }\left(t\right)$$

where $A\left(t\right)$ and $B\left(t\right)$ denote the system Jacobian matrices. To achieve zero-error tracking of the given reference trajectory, an integral term of the state error variable is introduced, and the equation is extended accordingly as follows:

(23) $$x_{\xi }\left(t\right)=\left[\begin{array}{c} \tilde{x}\left(t\right)\\ \tilde{\dot{x}}\left(t\right)\\ \int \tilde{x}\left(t\right)dt\\ \tilde{y}\left(t\right)\\ \tilde{\dot{y}}\left(t\right)\\ \int \tilde{y}\left(t\right)dt\\ \tilde{z}\left(t\right)\\ \tilde{\dot{z}}\left(t\right)\\ \int \tilde{z}\left(t\right)dt \end{array}\right]=\left[\begin{array}{c} x\left(t\right)-x_{r}\left(t\right)\\ x\left(t\right)-{\dot{x}}_{r}\left(t\right)\\ \int \left(x\left(t\right)-x_{r}\left(t\right)\right)dt\\ y\left(t\right)-y_{r}\left(t\right)\\ \dot{y}\left(t\right)-{\dot{y}}_{r}\left(t\right)\\ \int \left(y\left(t\right)-y_{r}\left(t\right)\right)dt\\ z\left(t\right)-z_{r}\left(t\right)\\ \dot{z}\left(t\right)-{\dot{z}}_{r}\left(t\right)\\ \int \left(z\left(t\right)-z_{r}\left(t\right)\right)dt \end{array}\right]$$

based on the Euler method, the deviation prediction model can be represented as the following discrete-time calibrated system:

(24) $$x_{\xi }\left(k+1\right)=\bar{A}\left(k\right)\cdot x_{\xi }\left(k\right)+\bar{B}\left(k\right)\cdot {\tilde{u}}_{\xi }\left(k\right)$$

The state variable $x_{\xi }\left(t\right)$ is decomposed into motion control in the z direction and motion control in the x and y directions, i.e.,

(25) $$x_{z}\left(k+1\right)={\bar{A}}_{z}\left(k\right)\cdot x_{\xi z}\left(k\right)+{\bar{B}}_{z}\left(k\right)\cdot {\tilde{u}}_{\xi z}\left(k\right)$$

(26) $$x_{xy}\left(k+1\right)={\bar{A}}_{xy}\left(k\right)\cdot x_{\xi xy}\left(k\right)+{\bar{B}}_{xy}\left(k\right)\cdot {\tilde{u}}_{\xi xy}\left(k\right)$$

where

(27) $${\bar{A}}_{z}=\left[\begin{array}{ccc} 1 & \Delta t & 0\\ 0 & 1 & 0\\ \Delta t & 0 & 1 \end{array}\right],\;{\bar{B}}_{z}\left[\begin{array}{c} 0\\ \frac{\Delta t}{m}\cos\theta \left(k\right)\cos\varphi \left(k\right)\\ 0 \end{array}\right]$$

(28) $${\bar{A}}_{xy}=\left[\begin{array}{cccccc} 1 & \Delta t & 0 & 0 & 0 & 0\\ 0 & 1 & 0 & 0 & 0 & 0\\ \Delta t & 0 & 1 & 0 & 0 & 0\\ 0 & 0 & 0 & 1 & \Delta t & 0\\ 0 & 0 & 0 & 0 & 1 & 0\\ 0 & 0 & 0 & \Delta t & 0 & 1 \end{array}\right]$$

(29) $${\bar{B}}_{xy}=\left[\begin{array}{cc} 0 & 0\\ \frac{\Delta t}{m}U_{1}\left(k\right) & 0\\ 0 & 0\\ 0 & 0\\ 0 & \frac{\Delta t}{m}U_{1}\left(k\right)\\ 0 & 0 \end{array}\right]$$

For the altitude subsystem in the z direction, the cost function of the MPC controller is formulated as shown in Equation (30):

(30) $$J_{z}={\left[\begin{array}{c} {\hat{x}}_{\xi z}-{\hat{x}}_{\xi rz} \end{array}\right]}^{T}Q_{z}\left[\begin{array}{c} {\hat{x}}_{\xi z}-{\hat{x}}_{\xi rz} \end{array}\right]+{\left[{\hat{\tilde{u}}}_{\xi z}-{\hat{\tilde{u}}}_{\xi rz}\right]}^{T}R_{z}\left[{\hat{\tilde{u}}}_{\xi z}-{\hat{\tilde{u}}}_{\xi rz}\right]$$

where $Q_{z}$ and $R_{z}$ represent the weighting matrices for the state variables and control inputs, respectively, and are both positive definite diagonal matrices, and $Q_{z}\in R^{N_{z}\times N_{z}}$,${\;R}_{z}\in R^{N_{u}\times N_{u}}$, and $N_{z}$ denotes the prediction horizon and $N_{u}$ the control horizon. The state prediction vector is defined as ${\hat{x}}_{\xi z}≜[{\hat{x}}_{\xi z}^{T}(k+1|k)\cdots {\hat{x}}_{\xi z}^{T}(k+N_{z}|k){]}^{T}$, and the control input vector is defined as ${\hat{u}}_{\xi z}≜[{\hat{u}}_{\xi z}^{T}(k|k)\cdots {\hat{u}}_{\xi z}^{T}(k+N_{u}-1|k){]}^{T}$. The predicted discrete-time state trajectory can be computed as follows:

(31) $$\begin{array}{c} {\hat{x}}_{\xi z}\left(k+1|k\right)=P_{Z}\left(k|k\right)\cdot x_{\xi z}\left(k|k\right) \end{array}+H_{Z}\left(k|k\right)\cdot {\hat{\tilde{u}}}_{\xi z}\left(k|k\right)$$

where ${\tilde{u}}_{\xi z}\left(k|k\right)=U_{1}\left(k\right)-U_{1r}\left(k\right)$. The reference deviation state vector and the reference deviation control vector are defined as follows:

(32) $${\hat{x}}_{\xi rz}=\left[\begin{array}{c} x_{\xi rz}\left(k+1\right)|k-x_{\xi rz}\left(k|k\right)\\ \vdots \\ x_{\xi rz}\left(k+N_{z}-1\right)|k-x_{\xi rz}\left(k|k\right) \end{array}\right]$$

(33) $${\hat{u}}_{\xi rz}=\left[\begin{array}{c} U_{1r}\left(k|k\right)-U_{1r}\left(k-1|k\right)\\ \vdots \\ U_{1r}\left(k+N_{u}-1|k\right)-U_{1r}\left(k|k\right) \end{array}\right]$$

By optimizing the performance index $J_{z}$, the control input for the deviation model of the altitude controller can be obtained as:

(34) $$\begin{array}{c} {\hat{\tilde{u}}}_{\xi z}={\left[{H_{z}}^{T}Q_{z}H_{z}+R_{z}\right]}^{-1}\cdot \end{array}\left[{H_{z}}^{T}Q_{z}\left({\hat{x}}_{\xi rz}\left(k\right)-P_{z}{\hat{x}}_{\xi z}\left(k\right)\right)+R_{z}{\hat{\tilde{u}}}_{\xi rz}\right]$$

Under the influence of environmental disturbances and noise, the compensation value Δu is defined as the particle of the PSO population. The fitness function is designed in the following form:

(35) $$f={\left(e_{k}\right)}^{2}+\lambda \cdot {\left(\Delta U_{1}\right)}^{2}+Penalty\left(g\right)$$

where $e_{k}=z_{r}\left(t\right)-z\left(t\right)$ represents the tracking error in the z direction. The term λ denotes the weight coefficient of the compensation input, defined as $\lambda =\lambda_{0}\cdot \left(1+{\dot{e}}_{k}\right)$, which allows for dynamic adjustment of the weight. This design prioritizes error suppression by reducing the weight of the compensation input when the error variation rate is large. $Penalty\left(g\right)$ is the constraint penalty term, defined as follows: $Pendty(g)=\sum (max(0,\nu_{z}-\nu_{zmax}){)}^{2}+\sum (max\left(0,U_{1}-U_{1max}\right){)}^{2}$. To prevent the control compensation from exceeding the maximum performance limits of the UAV. The update rule is defined as:

- If $f_{i}\left(j\right)<f\left(pbest_{i}\right)$, then update $pbest_{i}\leftarrow \Delta u_{i}\left(j\right)$
- If $f_{i}\left(j\right)<f\left(gbest_{i}\right)$, then update $gbest_{i}\leftarrow \Delta u_{i}\left(j\right)$

The control compensation for the altitude sub model is iteratively optimized to obtain the optimal compensation input $\Delta u_{z}$, expressed as:

(36) $${\hat{\tilde{u}}}_{z}={\hat{\tilde{u}}}_{\xi z}+\Delta u_{z}$$

Accordingly, the control input $U_{1}\left(K\right)$ is given by:

(37) $$U_{1}\left(K\right)=U_{1r}\left(k\right)+{\hat{\tilde{u}}}_{z}\left(k|k\right)$$

Similarly, for the x and y sub-systems, the control inputs $u_{x}$ and $u_{y}$ can be derived as follows:

(38) $$\left\{\begin{array}{c} u_{x}(k)=u_{xr}(k)+{\hat{\tilde{u}}}_{\xi x}+\Delta u_{x}\\ u_{y}(k)=u_{yr}(k)+{\hat{\tilde{u}}}_{\xi y}+\Delta u_{y} \end{array}\right.$$

Based on Equations (18) and (19), the reference Euler angles of the quadrotor UAV at the current time can be inversely calculated as follows:

(39) $$\left\{\begin{array}{l} \phi_{r}(k)=\arcsin(u_{x}(k)\sin\psi (k)-u_{y}(k)\cos\psi (k))\\ \theta_{r}\left(k\right)=\arcsin(\left(u_{x}\left(k\right)\cos\psi \left(k\right)+\frac{u_{y}\left(k\right)\cos\psi \left(k\right)}{\cos\phi_{r}\left(k\right)}\right) \end{array}\right.$$

The complete decision-making process of the proposed PSMC position controller is presented in Algorithm 2. The method combines an MPC-based nominal control law with a PSO-driven compensation loop for improved tracking robustness under dynamic disturbances.
**Algorithm 2.** Execution Procedure of the PSMC Position ControllerInput: Current UAV state $\gamma_{k}\;=\;{\left[x_{k},{\;y}_{k},\;\;z_{k}\right]}^{T}$            Desired reference $y_{d,k}$,            System matrices A, B            MPC horizons: prediction horizon N, control horizon M            Weighting matrices Q, R            Input and state constraints            PSO parameters Compute tracking error: $e_{k}\;\leftarrow \;y_{k}-\;y_{d,k}$ Linearize nonlinear UAV dynamics at operating point $y_{k}$:             Obtain linear system: $x_{k+1}=\;A\;x_{k}+\;B\;u_{k}$ Construct standard MPC cost function:             $J(u)\;=\sum_{i=0}^{n-1}\left[\|e_{k+i}\|Q^{2}\;+\;\|u_{k+i}\|R^{2}\right]\;$ Formulate MPC constraints:            State limits: $\;y_{min}\leq \;y_{k+i}\leq \;y_{max}$            Input limits: ${u\;}_{min}\leq \;u_{k+i}\;\leq \;u_{max}$                Terminal condition: optional Solve convex MPC optimization problem to obtain $\;u_{MPC}=\;[u_{k}\ldots ,{\;u}_{K+M-1}]$ Initialize PSO search around $u_{MPC[0]}$:             Define particle positions as perturbations $\Delta u\;\in \;R^{m}$ around $u_{MPC[0]}$ Define PSO objective function:             ${\;J}_{PSO(\Delta u)\;}=\;\|y_{predk\;+1}\;-\;y_{d,k+1}\|Q\;+\;\lambda {\left\|\Delta u\right\|}^{2}$ Run PSO optimizer (see Algorithm 1) to minimize ${\;J}_{PSO(\Delta u)\;}$             Obtain best particle ${\Delta u}_{opt}$ Compute final control input:             $u_{k}^{*}\leftarrow u_{MPC[0]}\;+\;{\Delta u}_{opt}$ Apply $u_{k}^{*}$ to UAV system Wait for next sampling interval and repeat Output: Optimized control input $u_{k}^{*}$

### 3.3. LQR Inner-Loop Controller

While the outer-loop controller governs the position-level behavior of the quadrotor, the attitude dynamics require a fast and stable response to ensure accurate tracking and control allocation. To this end, LQR is employed in the inner loop. However, conventional fixed-gain LQR may become suboptimal when the flight state varies significantly. Therefore, gain scheduling is incorporated to adjust the feedback gains according to the real-time operating conditions, and a control-rate relaxation mechanism is adopted to prevent aggressive input changes and reduce actuator saturation. These enhancements improve responsiveness, maintain stability, and reduce control effort under complex flight scenarios.

In this framework, the inner-loop controller stabilizes the rotational dynamics of the quadrotor across the roll, pitch, and yaw axes. Although LQR provides an analytical and optimal solution for linearized attitude regulation, its performance degrades when operating conditions vary or perturbations occur. Therefore, the enhanced design adopted in this work integrates gain scheduling and control-rate relaxation to improve adaptability and ensure smooth actuation. The attitude dynamics of the quadrotor are governed by rigid-body Euler equations. Let $\eta =[\phi ,\theta ,\psi {]}^{T}$ denote roll, pitch, and yaw angles, and $\omega =[\omega_{x},\omega_{y},\omega_{z}{]}^{T}$ represent body-frame angular velocities. The rotational dynamics can be modeled as:

(40) $$\dot{\eta }=J\left(\eta \right)\omega$$

(41) $$I\dot{\omega }=\tau -\omega \times \left(I\omega \right)$$

where I is the moment of inertia matrix and τ is the control torque vector. For small-angle maneuvers (hovering or slow transitions), the attitude system can be approximated by linearization around the hover condition (η≈0,  ω≈0), resulting in the decoupled form:

(42) $$\left\{\begin{array}{c} \dot{\phi }=\omega_{x},\;{\dot{\omega }}_{x}=\frac{1}{I_{x}}\tau_{\phi }\\ \dot{\theta }=\omega_{y},\;{\dot{\omega }}_{y}=\frac{1}{I_{y}}\tau_{\theta }\\ \dot{\psi }=\omega_{z},\;{\dot{\omega }}_{z}=\frac{1}{I_{z}}\tau_{\psi } \end{array}\right.$$

Each channel can thus be represented as a second-order linear system. For compactness, the general state-space form is written as:

(43) $${\dot{x}}_{r}=A_{r}x_{r}+B_{r}u_{r}$$

Its state and input vectors are:

(44) $$x_{r}=\left[\begin{array}{c} \eta -\eta_{ref}\\ \omega -\omega_{ref} \end{array}\right],\;u_{r}=\tau =\left[\begin{array}{c} \tau_{\phi }\\ \tau_{\theta }\\ \tau_{\psi } \end{array}\right]$$

The matrices $A_{r}$ and $B_{r}$ are block-diagonal:

(45) $$A_{r}=\left[\begin{array}{cc} 0 & I_{3}\\ 0 & 0 \end{array}\right],\;B_{r}=\left[\begin{array}{c} 0\\ I^{-1} \end{array}\right]$$

To enhance the smoothness and robustness of the LQR controller under noise or discrete implementation, we introduce a relaxation term penalizing the rate of change in the state, i.e., $∥{\dot{x}}_{r}{∥}^{2}$. The cost function is defined as:

(46) $$J=\int_{0}^{\infty }\;\left(x_{r}^{T}Qx_{r}+u_{r}^{T}Ru_{r}+\varepsilon \cdot {\dot{x}}_{r}^{T}{\dot{x}}_{r}\right)dt$$

where Q>0 penalizes attitude tracking errors, R>0 penalizes control efforts, ε≥0 is a relaxation factor controlling the sensitivity to rapid state transitions.

Let us denote the auxiliary term:

(47) $${\dot{x}}_{r}=A_{r}x_{r}+B_{r}u_{r}\Rightarrow {\dot{x}}_{r}^{T}{\dot{x}}_{r}=x_{r}^{T}A_{r}^{T}A_{r}x_{r}+2x_{r}^{T}A_{r}^{T}B_{r}u_{r}+u_{r}^{T}B_{r}^{T}B_{r}u_{r}$$

We substitute it into the cost function:

(48) $$J=\int_{0}^{\infty }\;\left[x_{r}^{T}\left(Q+\epsilon A_{r}^{T}A_{r}\right)x_{r}+u_{r}^{T}\left(R+\varepsilon B_{r}^{T}B_{r}\right)u_{r}+2\varepsilon x_{r}^{T}A_{r}^{T}B_{r}u_{r}\right]dt$$

The cross term $x_{r}^{T}A_{r}^{T}B_{r}u_{r}$ can be absorbed using matrix completion or treated as bounded coupling. The modified Riccati equation becomes:

(49) $$A_{r}^{T}P+PA_{r}-PB_{r}(R+\varepsilon B_{r}^{T}B_{r}{)}^{-1}B_{r}^{T}P+Q+\varepsilon A_{r}^{T}A_{r}=0$$

The optimal control law is:

(50) $$u_{r}=-K_{r}x_{r},K_{r}=(R+\varepsilon B_{r}^{T}B_{r}{)}^{-1}B_{r}^{T}P$$

This relaxed formulation allows tuning of the trade-off between response speed and smoothness and effectively attenuates chattering or high-frequency fluctuations from the outer-loop MPC.

To further improve performance under time-varying dynamics, we introduce a gain scheduling mechanism that dynamically adjusts the weight matrix Q based on the current state energy:

(51) $$Q\left(k\right)=Q_{0}\cdot \left(1+\rho \cdot ∥x_{r}\left(k\right){∥}^{2}\right)$$

where $Q_{0}$ is the nominal state penalty, ρ>0 is a tuning coefficient. This adaptive weighting increases the control stiffness when the system deviates significantly from the reference, ensuring rapid convergence without sacrificing nominal smoothness. To avoid computational burden, this scheme is implemented as a simple scalar scaling applied to the diagonal entries of Q, avoiding full Riccati precomputation at each step.

The LQR controller was tuned following a trial-and-error procedure guided by the physical meaning of the weighting matrices. Specifically, larger diagonal entries of Q penalize deviations in angular states, while larger entries of R penalize excessive control inputs. We first set Q as a diagonal matrix with values in the range [10, 200] for attitude states and [1, 20] for angular rates, and R as a diagonal matrix with values in [0.01, 1.0]. These values were adjusted iteratively until a satisfactory compromise between fast response and limited control effort was obtained. The final selected values are given in Section 5.1.

In addition, gain scheduling was introduced by scaling the diagonal terms of Q with respect to the reference velocity norm. For low-speed flight (<1.0 m/s), the baseline Q was used, while for higher speeds (>3.0 m), the diagonal terms were scaled by a factor of 1.4, with linear interpolation in between. This scheduling allowed the controller to maintain robustness at higher dynamic loads without excessive overshoot.

## 4. Lyapunov-Based Stability Analysis

To verify the theoretical stability of the proposed hierarchical PSMC–LQR control framework, Lyapunov stability analysis is conducted in this section. Based on the control laws derived in Section 3, the outer-loop PSMC–MPC generates desired attitude angles and thrust commands, while the inner-loop LQR controller stabilizes the attitude dynamics through torque regulation. To rigorously link the proposed control laws to the system’s stability, the composite error dynamics of the quadrotor are first formulated.

Let $e_{p}=[e_{x},e_{y},e_{z}{]}^{T}$ denote the position tracking error, and $e_{a}=[e_{\phi },e_{\theta },e_{\psi }{]}^{T}$ denote the attitude tracking error. According to Equations (9) and (10), the closed-loop error dynamics can be expressed as:

(52) $$\dot{e}=A_{e}+B_{e}∆u_{PSO}$$

where $e=[e_{p}^{T},e_{a}^{T}{]}^{T}$ represents the composite error vector, $A_{e}$ and $B_{e}$ are the system matrices, and $\Delta u_{PSO}$ is the bounded adaptive term introduced by the PSO compensator.

To facilitate the stability proof, we define a Lyapunov candidate function as:

(53) $$V=\frac{1}{2}e_{p}^{T}p_{1}e_{p}+\frac{1}{2}e_{a}^{T}p_{1}e_{a}$$

where $P_{1},{\;P}_{2}>0$ are symmetric positive-definite matrices corresponding to the position and attitude subsystems, respectively.

Taking the time derivative of V yields:

(54) $$\dot{V}=e_{p}^{T}P_{1}{\dot{e}}_{p}+e_{a}^{T}P_{2}{\dot{e}}_{a}=e^{T}(PA_{e}+A_{e}^{T}P)e+2e_{p}^{T}P_{1}B_{e}\Delta u_{PSO}$$

The first term in Equation (54) corresponds to the nominal linearized dynamics of the cascaded system, while the second term represents the perturbation caused by the PSO adaptive compensation.

To guarantee convergence, the feedback gain matrices of both the PSMC and LQR controllers are designed such that:

(55) $$PA_{e}+A_{e}^{T}P=-Q$$

where Q is a symmetric positive-definite matrix. Hence, the Lyapunov derivative becomes:

(56) $$\dot{V}=-e^{T}Qe+2e_{p}^{T}P_{1}B_{e}\Delta u_{PSO}$$

Since the PSO-based adaptive term $\Delta u_{PSO}$ is bounded and continuously adjusted to minimize the MPC cost, it satisfies:

(57) $$|2e_{p}^{T}P_{1}B_{e}\Delta u_{PSO}|\leq \varepsilon ∥e_{p}{∥}^{2}$$

where ε>0 is a sufficiently small constant. Substituting this inequality yields:

(58) $$\dot{V}\leq -(\lambda_{min}\left(Q\right)-\varepsilon )∥e{∥}^{2}$$

Therefore, as long as $\lambda_{min}(Q)>\varepsilon$, V is strictly decreasing, ensuring that the equilibrium point e=0 is asymptotically stable. This result confirms that the proposed PSMC–LQR framework guarantees closed-loop stability while enhancing robustness and adaptability.

## 5. Simulation and Discussion

To validate the effectiveness of the proposed control framework, this section presents a series of representative simulation experiments that evaluate the quadrotor’s trajectory tracking accuracy and robustness against external disturbances. In addition to performance evaluation, a detailed computational complexity analysis is also conducted to compare the algorithmic efficiency of the proposed PSMC-LQR controller with two baseline methods. This comprehensive assessment aims to demonstrate both the control effectiveness and real-time feasibility of the proposed scheme.

All simulations were implemented in Python 3.10.13 using the nonlinear quadrotor model introduced in Section 2. The controller sampling time was set to 20 ms, and the total simulation duration was fixed at 20 s for all test scenarios. A three-dimensional helical trajectory was used to evaluate position–attitude tracking performance under coupled dynamics. To emulate realistic perturbations, time-varying sinusoidal disturbances were applied along the x-, y-, and z-axes. The tracking performance is evaluated using four commonly adopted metrics: mean absolute error (MAE), root mean square error (RMSE), maximum tracking error (MaxErr), and control effort. Their explicit definitions and calculation formulas are provided in Section 5.3 and are not repeated here for conciseness.

To ensure rationality and fairness of comparison, all evaluated controllers shared the same quadrotor dynamics, initial conditions, sampling period, state constraints, and disturbance settings. We let the system be defined as $\dot{x}=f\left(x,u\right),\;\;x\left(0\right)=x_{0},{\;T}_{s}=constant$, and the same reference trajectory was used for all configurations. In addition, the weighting matrices of the baseline MPC and LQR, $Q_{p},\;\;R_{p}$ and $Q_{a},{\;R}_{a}$, were fixed across all compared methods unless one of the associated modules was intentionally deactivated in the ablation setting.

Parameter optimality was ensured through iterative tuning under the baseline architecture (MPC–LQR), where a grid-based adjustment of Q–R ratios and constraint bounds was performed to minimize the steady-state tracking error and control effort. Once the baseline weights achieved convergence and no further improvement was observed, the same parameter set was inherited by the other variants to eliminate bias from re-tuning. As a result, performance differences among methods arose solely from the activation of individual modules rather than from inconsistent parameter choices.

### 5.1. Trajectory Tracking Performance Evaluation in Calm Air Conditions

This subsection evaluates the performance of the proposed PSMC-LQR controller in tracking a helical trajectory under disturbance-free conditions. The helical trajectory and its parameters, which are specified below, are newly defined for this experiment and differ from those used in previous sections. The controller’s tracking performance is analyzed in terms of precision, error behavior, and response stability to demonstrate its effectiveness in ideal conditions.

This example first verifies the trajectory tracking performance of the quadrotor UAV in the absence of external disturbances. The parameters used in the simulation experiments are listed in Table 1 for the UAV model. The reference trajectories for the UAV in the x, y, and z directions are defined as $x_{ref}=5\sin(\frac{\pi t}{5})$,${\;y}_{ref}=5\cos(\frac{\pi t}{5})$,${\;z}_{ref}=0.5t$, and $\psi_{ref}=0$. The initial position and attitude of the quadrotor are given by $(x_{0}\;y_{0}\;z_{0}{)}^{T}=(0\;0\;0{)}^{T}m;\;(\phi_{0}\;\theta_{0}\;\psi_{0}{)}^{T}=(0\;0\;0{)}^{T}rad$. In all the simulations reported in this paper the desired yaw angle is kept constant (zero) to simplify the outer-loop/inner-loop decoupling and to isolate the performance of the position controller under structured translational disturbances. This is a common experimental choice in trajectory tracking comparisons where yaw control is orthogonal to position-control design.

To ensure reliable performance, all controller parameters were determined through iterative simulations and parametric analysis to balance trajectory accuracy, robustness, and computational efficiency.

For the PSO-based compensator, the parameters were selected to guarantee rapid convergence within each sampling period while maintaining real-time feasibility. The particle number and maximum iteration count were set to i=10 and $n_{max}=8$, respectively. Comparative simulations showed that increasing either parameter beyond these values provided less than 3% improvement in tracking accuracy but caused more than 20% growth in computation time. The inertia weight range was defined as $\omega_{max}=0.9$ and $\omega_{min}=0.4$, while the acceleration coefficients were set to $c_{1}=0.8$ and $c_{2}=0.8$, providing a balanced exploration–exploitation capability and stable convergence under model uncertainties. The weighting factor was assigned as $\lambda_{0}=0.7$ to regulate the amplitude of the correction term, avoiding excessive adjustment during strong disturbances.

For the MPC controller, the sampling interval was set to Δt=0.02 s, consistent with the outer-loop update rate. The weighting matrices were defined as $Q_{z}=diag(40,1)$, $R_{z}=diag(1)$, $Q_{xy}=diag(40,40,1,1)$, and $R_{xy}=diag(1,1)$. These values were tuned through iterative trials to emphasize position accuracy while maintaining smooth control inputs. Parameter sweeps revealed that larger Q/R ratios improved steady-state precision but reduced robustness to modeling errors, whereas the selected configuration achieved the best trade-off between accuracy and stability.

For the inner-loop LQR controller, the sampling period was set to ΔT=0.02 s, and the weighting matrices were chosen as Q=diag(200,200,2,2) and R=diag(1,1,1). Simulation-based parametric studies showed that increasing the Q/R ratio accelerated response but induced overshoot, while smaller ratios slowed stabilization. The adopted values provided the most balanced transient performance with limited control effort.

All parameters were finalized after multiple rounds of simulation-based iteration and sensitivity verification. Moderate perturbations (±20%) in these values did not significantly affect tracking accuracy or system stability, confirming the robustness of the selected configuration.

**Figure 3:**
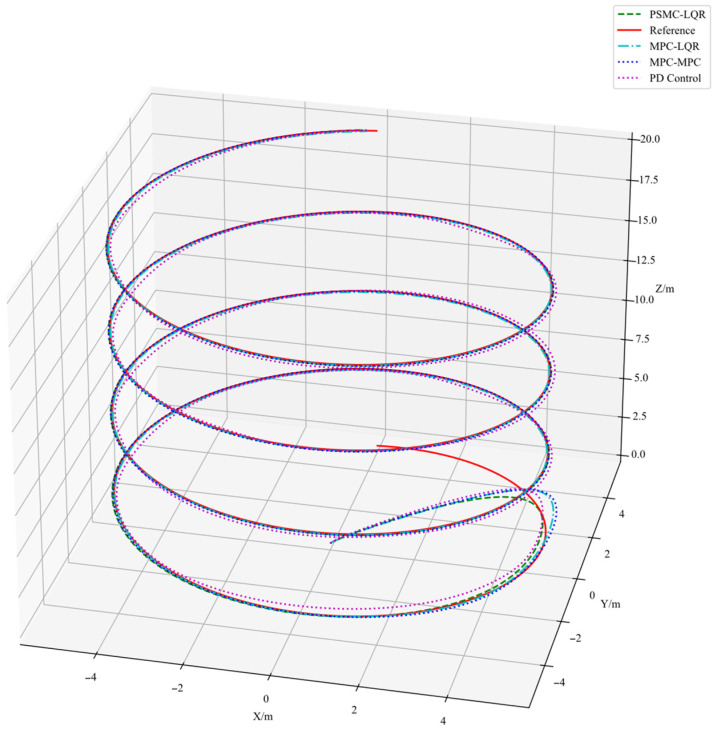
Helical Trajectory Tracking Results.

**Figure 4:**
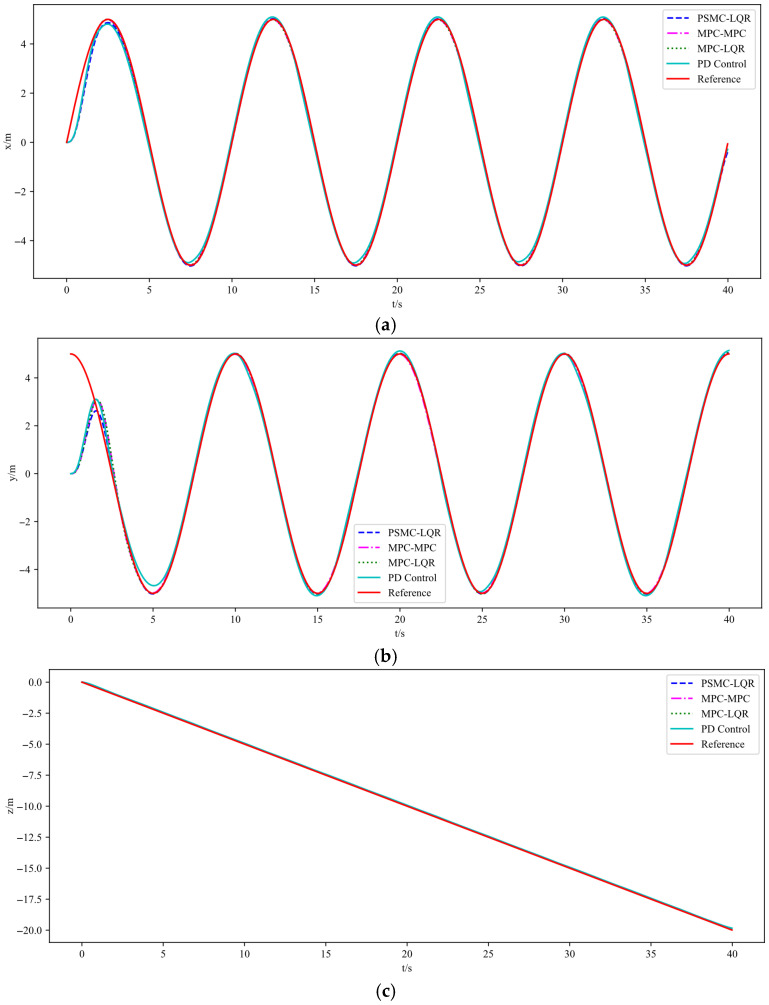
Tracking performance for different control strategies under ideal conditions. (**a**) Tracking performance along X axes. (**b**) Tracking performance along Y axes. (**c**) Tracking performance along Z axes.

**Figure 5:**
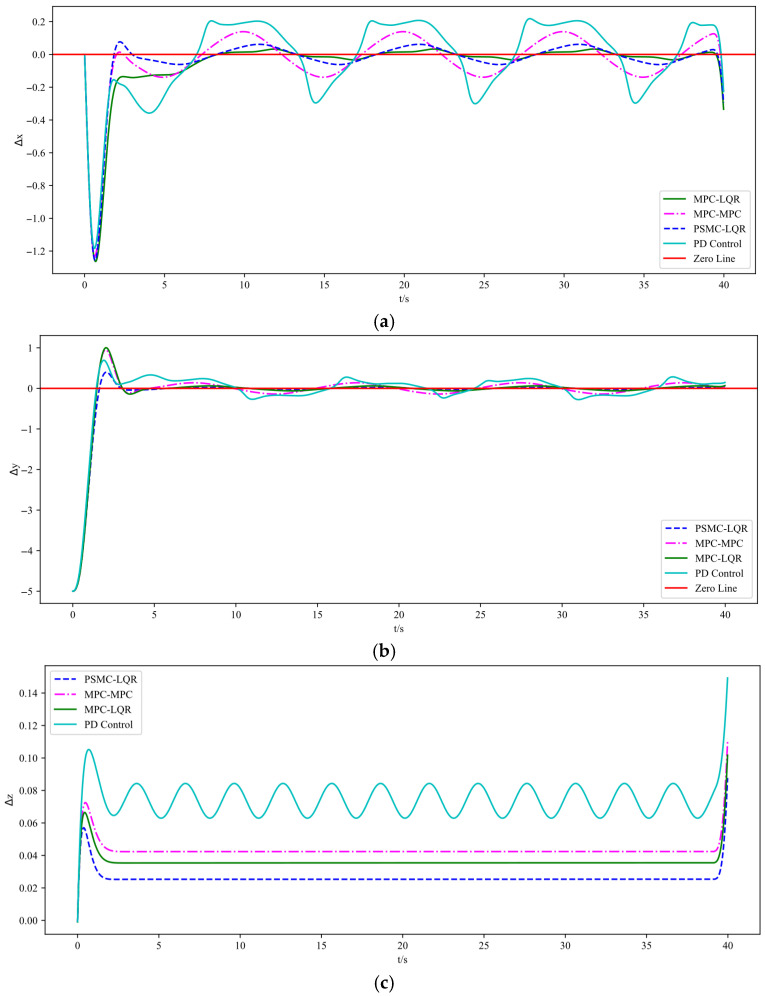
Tracking error curves under different control strategies. (**a**) Tracking error along X axes. (**b**) Tracking error along Y axes. (**c**) Tracking error along Z axes.

The simulation results are illustrated in Figure 3, Figure 4 and Figure 5. Specifically, Figure 3 depicts the UAV’s tracking performance along a helical trajectory, while Figure 4 shows the trajectory tracking curves along the x, y, and z axes and Figure 5 illustrates the tracking error curves in the three directions. In the figures, “MPC-MPC” denotes that both the outer and inner control loops adopt the MPC control strategy; “MPC-LQR” refers to the use of MPC in the outer loop and LQR in the inner loop; “PSMC-LQR” represents the novel control approach proposed in this study; and “PD Control” refers to a conventional proportional–derivative controller applied to both the outer and inner loops, serving as a representative of traditional control techniques. As observed from Figure 3, under ideal conditions without any external disturbances, all three control strategies enable the quadrotor UAV to accurately follow the desired trajectory, indicating that each control scheme possesses a certain degree of effectiveness in basic trajectory tracking performance.

As shown in Figure 3, under nominal conditions without external disturbances, all four control strategies enable the quadrotor UAV to follow the desired trajectory to some extent, demonstrating basic tracking feasibility. However, a closer inspection of Figure 4 reveals that the PD Control scheme exhibits the largest tracking deviation and oscillation due to its limited adaptability and lack of predictive capability. The MPC–MPC approach, serving as the baseline, achieves relatively stable tracking but still suffers from noticeable overshoot, while the MPC–LQR scheme exhibits minor undershoot. In contrast, the proposed PSMC–LQR controller achieves the smoothest and most accurate trajectory tracking by effectively suppressing overshoot and compensating for undershoot, and more importantly, it significantly outperforms the conventional PD Control scheme, highlighting its superiority over traditional control methods. Furthermore, the tracking error curves in Figure 5 confirm these findings quantitatively: the PSMC–LQR method yields the smallest steady-state error and lowest variance, followed by MPC–LQR and MPC–MPC, whereas the PD Control exhibits the largest cumulative tracking error, further verifying the robustness and precision advantages of the proposed hierarchical framework.

The experimental results indicate that the control scheme using MPC for both the outer and inner loops exhibits lower tracking accuracy compared to the combined MPC-LQR control method. The mean and variance of the tracking errors in each direction are presented in Table 2. Furthermore, in comparison with the MPC-LQR strategy, the proposed PSMC-LQR control method reduces tracking error by more than 13.2% in the x-direction, over 17.1% in the y-direction, and over 28% in the z-direction. In addition, when compared with the conventional PD control, which exhibits significantly larger errors and higher variances due to its lack of predictive and adaptive capabilities, the PSMC–LQR strategy demonstrates markedly superior accuracy and robustness. These results verify that the proposed hierarchical framework effectively suppresses disturbances, reduces steady-state deviations, and ensures smooth and precise trajectory tracking.

**Figure 6:**
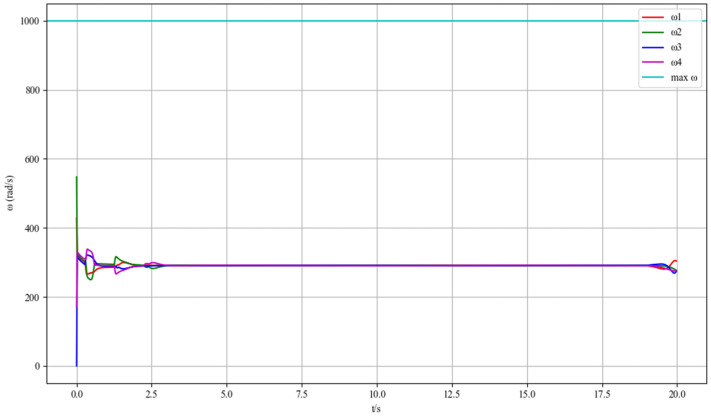
Time histories of the four motor speeds under calm conditions.

Figure 6 depicts the rotational speed responses of the four motors (ω1, ω2, ω3, ω4) of the UAV in a wind-free environment. At the initial moment (t = 0 s), the motors exhibit transient speed fluctuations, which originate from the UAV’s attitude-stabilizing process. The flight control system drives the motors rapidly adjust output torques, aiming to counteract initial attitude deviations and establish a stable flight posture. Within the first 2.5 s, the speeds of the four motors show complex yet coordinated variations. This transient behavior reflects the dynamic interaction between the motor control subsystem and the UAV’s inertial dynamics. After approximately 2.5 s, the speeds of all motors converge to a consistent stable value (around 300 rad/s). This steady—state convergence indicates that the control algorithm effectively suppresses transient disturbances in wind—free conditions, enabling the UAV to maintain a stable flight state with balanced motor outputs. Such results verify the basic performance of the motor control strategy: in a calm environment without external wind interference, the system can quickly stabilize the UAV’s attitude and maintain constant motor speeds, providing a reliable reference for trajectory tracking accuracy.

### 5.2. Trajectory Tracking Performance Evaluation in Wind Disturbance Conditions

This subsection evaluates the robustness of the proposed PSMC-LQR controller under time-varying external disturbances. By applying simulated wind forces in the x, y, and z directions, we emulate complex real-world conditions that UAVs typically encounter during outdoor missions. The same helical trajectory and controller configurations are retained to ensure experimental consistency. The performance of the three control methods is analyzed in terms of tracking precision, error resilience, and response stability.

To evaluate the robustness of the proposed controller against structured environmental perturbations, harmonic disturbances were introduced along the UAV’s translational axes. These disturbances were modeled as deterministic sinusoidal signals with distinct amplitudes and periods, representing periodic wind gusts or aerodynamic oscillations. This study focused on harmonic excitations as structured disturbances to clearly expose the disturbance rejection capabilities of the controllers, and the selection rationale was: (i) harmonic disturbances enable controlled and repeatable tests across different controllers; (ii) the amplitudes were chosen to represent light-to-moderate gusts that are typical for small quadrotors. For reproducibility, a short table detailing the magnitudes and units of relevant parameters is included here. External disturbances in the simulations were modeled as deterministic harmonic signals applied to translational dynamics: $F_{x}(t)=A_{x}sin(2\pi f_{x}t+\phi_{x})$, $F_{y}(t)=A_{y}sin(2\pi f_{y}t+\phi_{y})$, $F_{z}(t)=A_{z}sin(2\pi f_{z}t+\phi_{z})$, where A{x,y,z}, f{x,y,z}  and ϕ{x,y,z} denote amplitude, frequency and phase, respectively. The specific values used in the experiments reported in this paper are summarized in Table 3 below.

**Figure 7:**
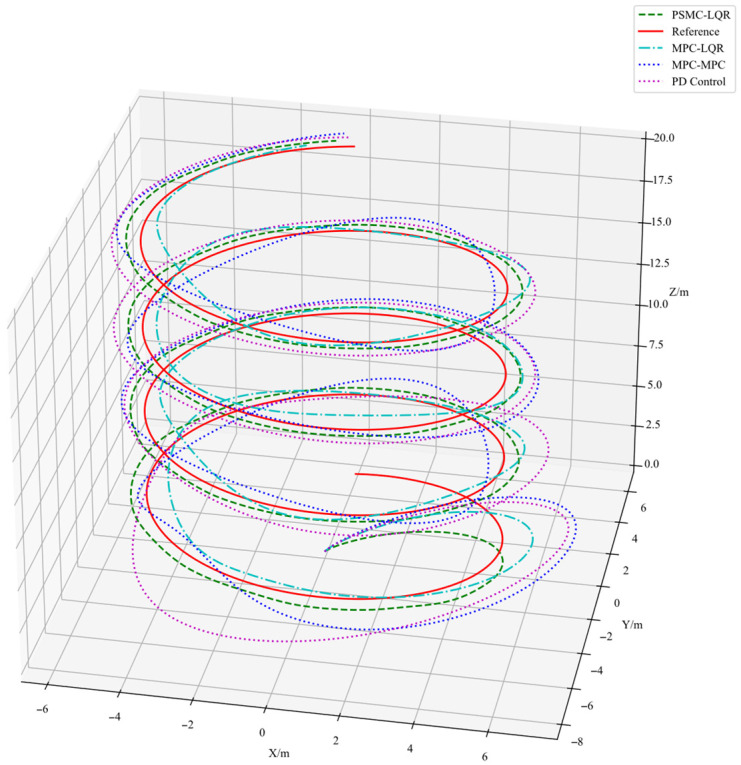
Helical Trajectory Tracking Results Under Wind Disturbance.

**Figure 8:**
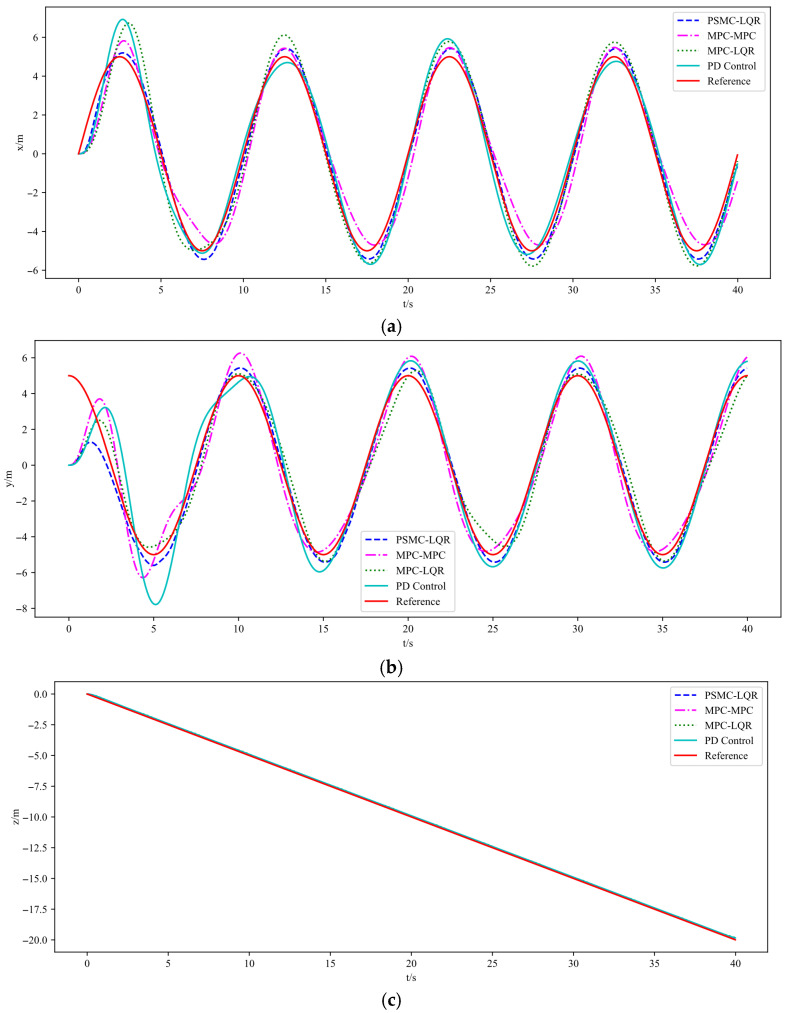
Trajectory tracking results under Wind Disturbance. (**a**) Tracking results along X axes. (**b**) Tracking results along Y axes. (**c**) Tracking results along Z axes.

**Figure 9:**
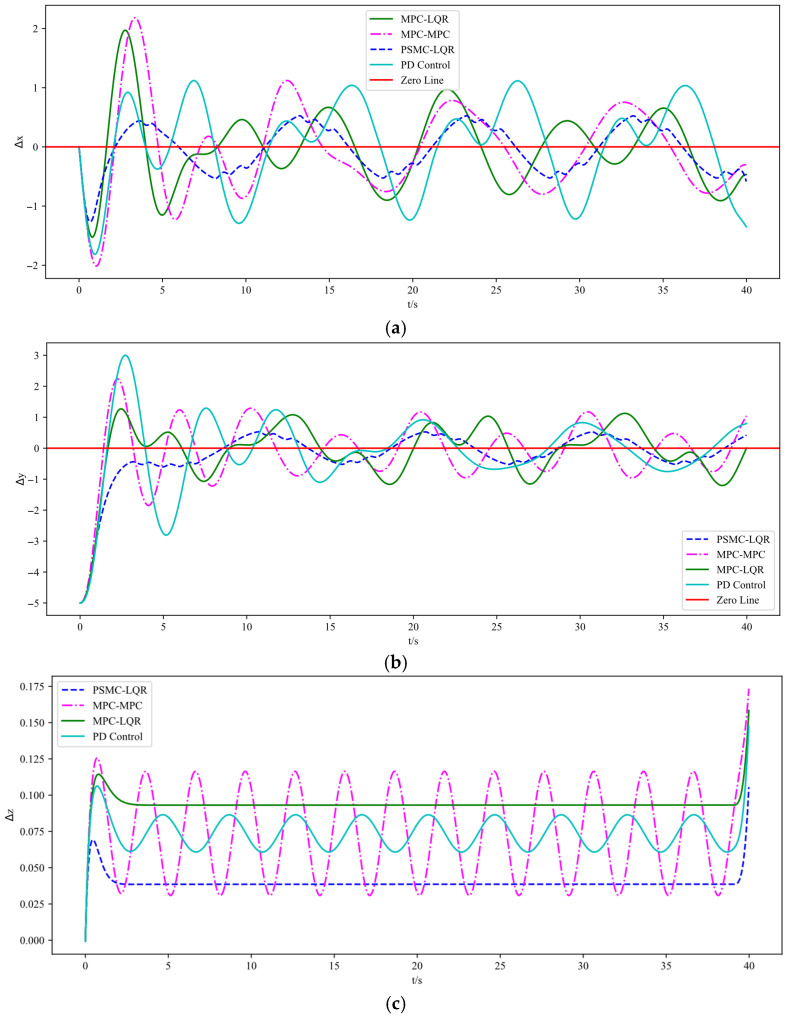
Tracking error curves under Wind Disturbance. (**a**) Tracking errors along X axes. (**b**) Tracking errors along Y axes. (**c**) Tracking errors along Z axes.

Figure 7 presents the spiral trajectory tracking results of the UAV under wind disturbances. Figure 8 shows the trajectory tracking curves in the x, y, and z directions under the same disturbance conditions, while Figure 9 depicts the corresponding tracking error curves in each direction. From the experimental results in Figure 7, Figure 8 and Figure 9, it is evident that the introduction of external disturbances leads to noticeable oscillations and deviations in the UAV’s flight trajectory. Among the four tested strategies, the PD Control exhibits the most significant tracking deviation due to its lack of predictive and adaptive capabilities. The MPC–MPC scheme maintains basic tracking performance but shows clear oscillatory behavior, while the MPC–LQR method provides improved stability yet still suffers from minor steady-state errors. In contrast, the proposed PSMC–LQR controller achieves the most stable and accurate trajectory tracking under wind disturbances, effectively suppressing oscillations and reducing trajectory deviations. These results clearly demonstrate the superior capability of the PSMC-LQR strategy in mitigating external disturbances and enhancing trajectory tracking performance for quadrotors.

The experimental results demonstrate that the proposed PSMC-LQR control method significantly improves trajectory tracking accuracy compared to both the hierarchical MPC control and the combined MPC-LQR control strategies. The mean and variance of the tracking errors in each direction under wind disturbance are presented in Table 4. Specifically, the PSMC-LQR method reduces the average tracking error in the x-direction by more than 34%, in the y-direction by over 26.2%, and in the z-direction by more than 46.8%. Despite wind disturbances, the system maintains stable flight. Furthermore, compared with the traditional PD Control, which exhibits large deviations and slow error recovery under external disturbances, the proposed framework demonstrates significantly enhanced disturbance rejection and robust trajectory tracking capability. These findings confirm that the PSMC–LQR strategy effectively suppresses wind-induced oscillations and ensures stable flight performance even in challenging conditions.

**Figure 10:**
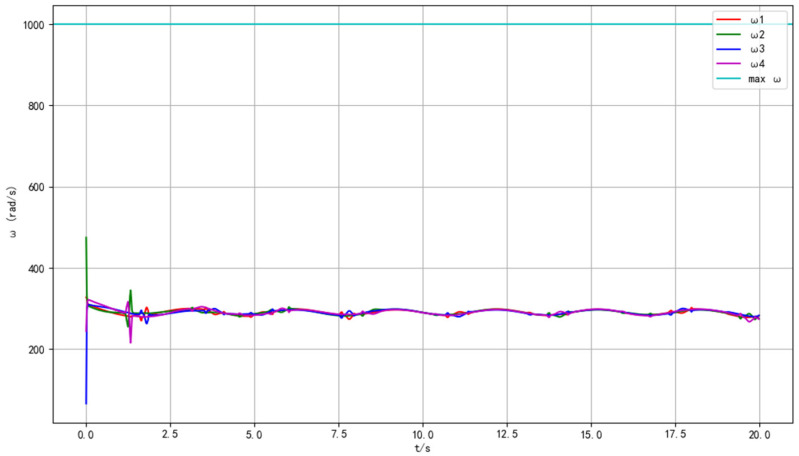
Time histories of the four motor speeds under wind disturbance.

Figure 10 illustrates the motor rotational speed characteristics of the UAV under wind-disturbed conditions. At t = 0 s, the speed deviations among the four motors are more significant compared to the wind—free case, directly reflecting the impact of wind on the UAV’s initial aerodynamic balance. In the transient phase (t < 2.5 s), the speeds of ω1–ω4 fluctuate violently: the wind disrupts the UAV’s attitude, and the flight control system triggers rapid motor speed adjustments to compensate for wind—induced disturbances (e.g., side-wind-caused lateral forces, gust-induced pitch changes). From 2.5 s to 20 s, although the motor speeds do not fully converge to an absolute constant value like in the wind—free scenario, they maintain relatively stable oscillations around a mean level (approximately 280–320 rad/s). This phenomenon demonstrates the adaptive regulation capability of the control system: facing continuous wind interferences, the algorithm dynamically allocates motor outputs to balance the UAV’s attitude and trajectory, rather than pursuing strict single—point speed stability. The minor speed oscillations are the result of the control system continuously correcting deviations caused by wind changes (such as wind speed fluctuations, direction shifts). These data highlight the robustness of the motor control scheme in complex environments: even under wind disturbances, the system can adjust motor speeds in real-time to ensure the UAV’s flight stability, laying a foundation for subsequent trajectory tracking tasks under dynamic wind conditions.

### 5.3. Ablation Analysis of Component Contributions

To further verify the contribution of each component in the proposed control framework, an ablation study was conducted under the same reference trajectory and disturbance settings. By selectively enabling or disabling the PSO-based compensator, gain scheduling, and control-rate relaxation, the individual and combined effects of each module could be quantitatively assessed. This design allows us to identify how each mechanism contributes to the overall tracking accuracy, robustness, and control effort.

To rigorously evaluate the role of each functional module in the proposed control architecture, an ablation study was performed using the same helical trajectory and disturbance conditions as in Section 6. Six controller variants were compared. The first is the baseline MPC–LQR, which employs an MPC-based outer loop and an LQR-based inner loop without any enhancement. The second adds the PSO-based compensator to the outer loop. The third incorporates gain scheduling in the inner loop, while the fourth enables only the rate relaxation mechanism. The fifth applies both gain scheduling and rate relaxation without PSO. The sixth corresponds to the full PSMC–LQR configuration, in which the PSO compensator, gain scheduling, and rate relaxation operate jointly.

Each configuration was evaluated with N = 10 Monte Carlo runs. In each run, the harmonic disturbances described in Section 5.2 were applied with randomized initial phases, and the same initial state and reference trajectory were used across configurations. The performance metrics reported for each run are: per-axis mean absolute error ${MAE}_{x}$, ${MAE}_{y}$, ${MAE}_{z}$, overall RMSE, maximum tracking error, control effort (mean absolute actuator command), and computational load (average and maximum per-step computation time in milliseconds). For a quantity q(t) computed over t = 1,…,T, MAE and RMSE were computed as:

(59) $${MAE}_{x}=\frac{1}{T}\sum_{t=1}^{T}\left|x\left(t\right)-x_{ref}\left(t\right)\right|$$

(60) $${MAE}_{y}=\frac{1}{T}\sum_{t=1}^{T}\left|y\left(t\right)-y_{ref}\left(t\right)\right|$$

(61) $${MAE}_{z}=\frac{1}{T}\sum_{t=1}^{T}\left|z\left(t\right)-z_{ref}\left(t\right)\right|$$

(62) $$MaxErr=\underset{t}{max}\sqrt{(x-x_{ref}{)}^{2}+(y-y_{ref}{)}^{2}+(z-z_{ref}{)}^{2}}$$

(63) $$RMSE=\sqrt{\frac{1}{T}\sum_{t=1}^{T}\left((x-x_{ref}{)}^{2}+(y-y_{ref}{)}^{2}+(z-z_{ref}{)}^{2}\right)}$$

(64) $$\overset{-}{\left|u\right|}=\frac{1}{T}\sum_{t=1}^{T}∥u(t){∥}_{1}$$

The numerical values reported in Table 5 were computed using the performance definitions in Equations (54)–(58). For each configuration, the mean absolute tracking errors along the x-, y- and z-axes (${MAE}_{x}$, ${MAE}_{y}$, ${MAE}_{z}$), the overall root mean square error (RMSE), the maximum instantaneous deviation (MaxErr), and the control effort were averaged over the Monte Carlo runs. These metrics provide complementary views of trajectory accuracy, robustness to perturbations, and actuation efficiency, allowing a fair comparison of the individual contributions from PSO compensation, gain scheduling, and rate relaxation.

The results in Table 5 indicate that each of the three components contributes to a distinct aspect of the closed-loop performance. The PSO-based outer-loop compensation slightly reduces steady-state deviations but does not substantially improve control expenditure on its own—this aligns with its core role of mitigating prediction mismatch in the outer loop, where it primarily refines position prediction accuracy rather than optimizing actuation efficiency. Inner-loop gain scheduling and rate relaxation, when enabled individually, yield noticeable improvements in vertical tracking accuracy and actuation smoothness under wind perturbations: specifically, gain scheduling enhances attitude convergence to adapt to varying flight conditions, while rate relaxation substantially decreases control expenditure and suppresses overshoot. Notably, the full PSMC–LQR configuration consistently achieves the lowest RMSE and MaxErr across all runs, while preserving one of the lowest control efforts among the tested settings. When all components operate in synergy, the proposed framework achieves the optimal overall balance in tracking precision, disturbance rejection and control smoothness. This confirms that the combined action of prediction-based compensation, scheduled feedback gains, and rate constraints leads to a more robust and accurate trajectory tracking control framework than any partial variant.

### 5.4. Computational Complexity Analysis

To quantitatively assess the computational characteristics of the proposed PSMC-LQR framework, a comparative complexity analysis is performed across three control architectures: the baseline MPC-MPC, the hybrid MPC-LQR, and the proposed PSO-augmented MPC-LQR scheme. The analysis focuses on per-step computational overhead, primarily considering the dominant operations involved—namely, the quadratic programming (QP) cost associated with MPC optimization and the metaheuristic search complexity introduced by the PSO-based compensation module.

MPC-MPC: At each time step, two sequential MPC optimizations are solved—first for trajectory generation and then again for attitude control. Assuming each MPC solver uses a standard quadratic programming (QP) solver with complexity $O(n^{3})$ for control horizon length n, the total complexity per step is approximately:

$$T_{MPC\text{-}MPC}=2\cdot O(n^{3})$$

MPC-LQR: Here only the outer-loop MPC is solved (complexity $O(n^{3})$, while the inner-loop LQR uses fixed-gain feedback with constant cost $O(n^{2})$. Hence:

$$T_{MPC\text{-}LQR}=O(n^{3})+O(n^{2})\approx O(n^{3})$$

PSMC-LQR: Combines the outer MPC solve $O(n^{3})$ with a bounded PSO search step. PSO complexity is  O(Np⋅Tp⋅m), where Np is particle count, Tp iteration count, and mmm control dimension. Since Np and Tp are kept small (e.g., Np≤20, Tp≤5), PSO’s overhead is approximately O(1) relative to MPC. Thus:

$$T_{PMPC\text{-}LQR}=O(n^{3})+O(1)\approx O(n^{3})$$

In summary, all three methods share the same cubic computational complexity in the MPC portion. Although PSMC-LQR adds a PSO-based adjustment step, the additional time is marginal compared to the MPC solve and remains constant per step. Importantly, simulation and embedded test-bed results confirm that PSMC-LQR achieves at least 50% improvement in trajectory tracking accuracy without increasing control cycle time or exceeding hardware real-time constraints.

## 6. Conclusions

This paper presented a hierarchical control framework for quadrotor trajectory tracking that combines a nonlinear model predictive controller with a PSO-based compensator in the outer loop (PSMC) and an LQR controller with rate relaxation and gain scheduling in the inner loop. The main motivation was to achieve robust real-time tracking performance under disturbances while retaining computational feasibility for embedded deployment. A discrete-time Lyapunov analysis was also conducted to support the stability of the proposed architecture. Through extensive simulations on helical trajectories with and without wind disturbances, the proposed PSMC–LQR achieved consistently better performance compared with the MPC–LQR and MPC–MPC baselines. In calm conditions, the mean tracking errors in the x, y, and z directions were reduced by more than 13.2%, 17.1%, and 28%, respectively. Under wind disturbances the reductions were more than 34%, 26.2%, and 46.8%, respectively. These results indicate that the hybrid use of MPC anticipation and PSO-based adaptability yields a good compromise between robustness and accuracy. The inclusion of rate relaxation and gain scheduling in the LQR inner loop further contributed to smoother attitude responses and reduced overshoot. The proposed control architecture highlights a practical way to integrate heuristic optimization (PSO) with model-based control (MPC + LQR). This demonstrates that embedding lightweight metaheuristics into MPC frameworks can significantly improve disturbance rejection while keeping the computational complexity within a range suitable for real-time implementation. The approach can be extended to other underactuated aerial vehicles or ground robots facing similar disturbance-prone environments. The current study is limited to simulation experiments with fixed yaw and structured harmonic disturbances. No onboard implementation results or computational benchmarks on embedded processors are reported at this stage. Moreover, only one reference trajectory (a helix) was tested; additional paths such as aggressive maneuvers or obstacle-rich trajectories could provide a broader evaluation.

Our next steps will include hardware-in-the-loop and onboard tests to quantify real-time computational load; extension of the framework to fully coupled yaw dynamics and variable yaw scenarios; experiments under stochastic turbulence models such as Dryden or von Kármán spectra; and exploration of adaptive or learning-based alternatives to PSO to further enhance online adaptability. These directions are expected to strengthen both the theoretical contribution and the practical applicability of the proposed method.

## Figures and Tables

**Table 1:** Quadrotor Model Parameters.

Parameter	Value
Mass	1.0 kg
Gravitational acceleration	$9.8\;m/s^{2}$
Moment of inertia about the X-axis	$4\times 10^{-3}\;{kgm}^{2}$
Moment of inertia about the Y-axis	$4\times 10^{-3}\;{kgm}^{2}$
Moment of inertia about the Z-axis	$8.4\times 10^{-3}\;{kgm}^{2}$

**Table 2:** Tracking Error Statistics under Wind-Free Conditions.

Metric	MPC-MPC	MPC-LQR	PSMC-LQR	PD Control
X-Error Variance	0.03586	0.03400	0.03067	0.05741
Y-Error Variance	0.47491	0.46824	0.46974	0.47777
Z-Error Variance	0.000031	0.000028	0.000025	0.000104
X-Mean Error	0.1117	0.0775	0.0673	0.1852
Y-Mean Error	0.2150	0.1746	0.1447	0.2719
Z-Mean Error	0.04334	0.03638	0.02621	0.7455

**Table 3:** Wind disturbance parameters used in simulations.

Disturbance	Amplitude A(m)	Frequency f(Hz)	Phase ϕ(rad)
$F_{x}$	5	2/15	0
$F_{y}$	4	2/15	0.5π
$F_{z}$	2	0.2	0.5π

**Table 4:** Tracking Error Statistics under Wind Disturbance.

Metric	MPC-MPC	MPC-LQR	PSMC-LQR	PD Control
X-Error Variance	0.55657	0.53966	0.14670	0.55657
Y-Error Variance	1.02599	0.90950	0.64590	1.35781
Z-Error Variance	0.00984	0.000045	0.000030	0.000126
X-Mean Error	0.6063	0.5109	0.3370	0.6103
Y-Mean Error	0.7352	0.6464	0.4768	0.7970
Z-Mean Error	0.0748	0.0938	0.0395	0.0743

**Table 5:** Comparison of Tracking Error and Control Performance in Ablation Experiments.

Method	${MAE}_{x}$	${MAE}_{y}$	${MAE}_{z}$	RMSE	MaxErr	Control Effort
MPC–LQR (baseline)	12.48	14.67	21.80	39.86	85.19	43.52
MPC–LQR + PSO	12.52	14.63	21.88	39.96	85.32	43.61
MPC–LQR + Scheduling only	12.40	14.72	22.32	40.17	84.89	43.81
MPC–LQR + Relaxation only	12.10	12.82	5.93	28.31	70.70	22.14
MPC–LQR (inner-only)	12.10	12.81	5.94	28.31	70.68	22.14
PSMC–LQR (full)	12.06	12.78	5.91	28.25	70.51	22.12

## Data Availability

Data are contained within the article.

## Appendix A

To facilitate the design of the outer-loop PSMC, the nonlinear dynamics of the quadrotor system are linearized around a time-varying reference trajectory and discretized using zero-order hold assumptions. This appendix provides the detailed mathematical procedure used for obtaining the linear discrete-time state-space model.

The full state vector is defined as:

(A1) $$x={\left[\begin{array}{cccccccccccc} p_{x} & p_{y} & p_{z} & v_{x} & v_{y} & v_{z} & \phi & \theta & \psi & \omega_{x} & \omega_{y} & \omega_{z} \end{array}\right]}^{T}$$

where $p_{i}$ are positions, $v_{i}$ are linear velocities, $\left(\phi ,\theta ,\psi \right)$ are Euler angles, and $\omega_{i}$ are angular rates.

The translational motion of the quadrotor can be expressed as:

(A2) $$\dot{p}=v$$

(A3) $$m\dot{v}=R\left(\phi ,\theta ,\psi \right){\left[\begin{array}{ccc} 0 & 0 & T \end{array}\right]}^{T}-mg{\left[\begin{array}{ccc} 0 & 0 & 1 \end{array}\right]}^{T}$$

where m is the quadrotor mass, T is total thrust, g is gravity, and R(⋅) is the body-to-inertial rotation matrix.

Linearization is performed using first-order Taylor expansion around the current operating point $\left(x_{ref},u_{ref}\right)$:

(A4) $$\delta \dot{x}=A_{c}\delta x+B_{c}\delta u$$

where $A_{c}={\left.\frac{\partial f}{\partial x}\right|}_{\left(x_{ref},u_{ref}\right)}$, $B_{c}={\left.\frac{\partial f}{\partial u}\right|}_{\left(x_{ref},u_{ref}\right)}$, $\delta x=x-x_{ref}$*,* $\delta u=u-u_{ref}$. Taking partial derivatives, the nonzero blocks of $A_{c}$ include:

(A5) $$\frac{\partial \dot{p}}{\partial v}=I_{3},\;\;\;\;\;\;\frac{\partial \dot{v}}{\partial \phi ,\theta }\approx \frac{T}{m}\cdot \frac{\partial R}{\partial \phi ,\theta }$$

where the approximation assumes small angles and thrusts T near hover. The matrix $B_{c}$ includes:

(A6) $$\frac{\partial \dot{v}}{\partial T}=\frac{1}{m}R\cdot e_{3}$$

where $e_{3}=[\begin{array}{ccc} 0 & 0 & 1 \end{array}{]}^{T}$. The continuous-time linear system is discretized using first-order matrix exponential:

(A7) $$A_{d}=e^{A_{c}\Delta t}\approx I+A_{c}\Delta t,\;\;\;\;\;B_{d}=\int_{0}^{\Delta t}\;e^{A_{c}\tau }d\tau \cdot B_{c}\approx B_{c}\Delta t$$

where Δt is the sampling time. This approximation holds well for fast-sampled quadrotor systems. The resulting discrete-time linear model

(A8) $$x_{k+1}=A_{d}x_{k}+B_{d}u_{k}$$

is used by the outer-loop MPC for trajectory prediction and control optimization. This model is re-linearized at each sampling instant along the reference trajectory.
